# Specific Endocytosis Blockade of *Trypanosoma cruzi* Exposed to a Poly-LAcNAc Binding Lectin Suggests that Lectin-Sugar Interactions Participate to Receptor-Mediated Endocytosis

**DOI:** 10.1371/journal.pone.0163302

**Published:** 2016-09-29

**Authors:** Sébastien Brosson, Frédéric Fontaine, Marjorie Vermeersch, David Perez-Morga, Etienne Pays, Sabrina Bousbata, Didier Salmon

**Affiliations:** 1 Laboratory of Molecular Parasitology, Institute of Molecular Biology and Medicine, Université Libre de Bruxelles, 12 rue des Professeurs Jeener et Brachet, B-6041 Gosselies, Belgium; 2 Institute of Medical Biochemistry Leopoldo de Meis, Centro de Ciências e da Saúde, Federal University of Rio de Janeiro, Av. Brigadeiro Trompowsky, Rio de Janeiro, 21941-590, Brazil; 3 Center for Microscopy and Molecular Imaging-CMMI, Université Libre de Bruxelles, 8 rue Adrienne Bolland, B-6041 Gosselies, Belgium; Tulane University, UNITED STATES

## Abstract

*Trypanosoma cruzi* is a protozoan parasite transmitted by a triatomine insect, and causing human Chagas disease in South America. This parasite undergoes a complex life cycle alternating between non-proliferative and dividing forms. Owing to their high energy requirement, replicative epimastigotes of the insect midgut display high endocytic activity. This activity is mainly restricted to the cytostome, by which the cargo is taken up and sorted through the endosomal vesicular network to be delivered to reservosomes, the final lysosomal-like compartments. In African trypanosomes tomato lectin (TL) and ricin, respectively specific to poly-*N*-acetyllactosamine (poly-LacNAc) and β-D-galactose, allowed the identification of giant chains of poly-LacNAc in *N*-glycoproteins of the endocytic pathway. We show that in *T*. *cruzi* epimastigote forms also, glycoproteins of the endocytic pathway are characterized by the presence of N-linked glycans binding to both ricin and TL. Affinity chromatography using both TL and *Griffonia simplicifolia* lectin II (GSLII), specific to non-reducing terminal residue of *N*-acetylglucosamine (GlcNAc), led to an enrichment of glycoproteins of the trypanosomal endocytic pathway. Incubation of live parasites with TL, which selectively bound to the cytostome/cytopharynx, specifically inhibited endocytosis of transferrin (Tf) but not dextran, a marker of fluid endocytosis. Taken together, our data suggest that *N*-glycan modification of endocytic components plays a crucial role in receptor-mediated endocytosis of *T*. *cruzi*.

## Introduction

*Trypanosoma cruzi* is the ethiological agent of Chagas’ disease, a parasitic disease affecting about 8 million individuals in Latin America [1]. The life cycle of this parasite involves two intermediate hosts (a triatomine insect vector and a vertebrate host) and three well-defined developmental stages: trypomastigote (non-replicative infectious form), amastigote (intracellular dividing form found in the vertebrate host) and epimastigote (replicative form found in the midgut of the insect vector). The latter form has endocytic activity, which is absent from the two other forms (reviewed in [2]). In *T*. *cruzi*, endocytosis is restricted to two specialized invaginations of the plasma membrane around the base of the flagellum: the flagellar pocket (FP), which is devoid of subpellicular microtubules, and the cytostome, which is linked to few special microtubules that penetrate deeply into the cell [3] (reviewed in [2] and [4]). The cytostome is observed only in epimastigote and amastigote forms and is absent from the related kinetoplastids *T*. *brucei* and *Leishmania*. As opposed to *T*. *brucei* bloodstream forms, in which the endocytic turnover from the FP area is exceptionally high [5, 6], in the insect forms of *T*. *cruzi* the endocytic rate is much lower and the cytostome is the major site of endocytosis [7, 8]. In these parasites, the cargo is taken up through a system of pleomorphic tubular and cisternal structures constituting the early endosomes, which localize near the FP. Afterwards, the endocytosed material is delivered through tubular-vesicular endosomes to terminal lysosomal-like organelles, termed reservosomes, which contain an electron-dense protein matrix with inner membranes and an electron-lucent lipid inclusions [2] accumulating mostly near the posterior end of the cell [8, 9]. These organelles represent the ultimate stage of the endocytic pathway wherein accumulate digestive enzymes (hydrolases) and crystalloid lipid inclusions of neutral lipids such as cholesterol, which the parasite is able to mobilize upon serum starvation [10]. In contrast to what occurs in African trypanosomes, endocytosis is not mediated via coated vesicles and seems to be mainly clathrin-independent and cholesterol-dependent [11–13], although *T*. *cruzi* also internalizes ligands (*e*. *g*., Tf) by receptor-mediated endocytosis [8, 11, 14, 15]. Contrarily to the heterodimeric TfR encoded by ESAG6/7 in *T*. *brucei*, the nature of the elusive TfR is still matter of debate in *T*. *cruzi*. Albeit the presence of a saturable TfR was suggested in amastigote stages, which possesses a Tf binding protein that cross-reacts with a band of around 200 KDa using an anti-human TfR, it is still unclear whether this protein is not merely a host contaminant [16]. Freeze fracture studies demonstrated that the plasma membrane between the cytostome and the FP is distinct from the rest of the plasma membrane and contains a glycocalyx-like structure that appears rough in high-resolution field emission SEM [2]. This peculiar aspect is due to the presence of a high concentration of surface glycoconjugates that strongly bind concanavalin A (ConA), but fail to bind lectins that label the rest of the plasma membrane, such as *Ricinus communis* agglutinin I (RCA-I) or *Wisteria floribunda* agglutinin (WFA) [17]. These observations resemble those made in African trypanosomes, where ConA-binding proteins are abundantly present in the FP of both stages of the parasite [18]. In contrast ricin-binding glycoproteins were found to bind exclusively to the anterior membrane of the FP (flagellar adhesion zone), while wheat germ agglutinin (WGA) was uniformly distributed throughout the cell surface, including the free flagellum and flagellar adhesion zone [19]. Moreover, the entire endocytic pathway of *T*. *brucei* contains giant poly-*N*-acetyllactosamine (poly-LacNAc) side chains recognized by tomato lectin (TL) and ricin [20]. TL binds with high affinity to trimers and tetramers of *N*-acetyllactosamine (Gal β1–4 GlcNAc) repeats [21]. TL has also been shown in lectin blot analysis to recognize different sugar chain units in complex-type and oligomannose-type *N*-glycans, in particular the residual Man_3_GlcNAc_2_ (Manα1–3(Manα1–6)Manβ1–4GlcNAcβ1–4GlcNAc) pentasaccharide of complex-type *N*-glycans sequentially treated with exoglycosidases (sialidase, *β*-galactosidase, and *β*-*N*-acetylhexosaminidase) and to the exposed ManGlcNAc_2_ (Manβ1–4GlcNAcβ1–4GlcNAc) trisaccharide core of oligomannose *N*-glycans treated with *α*-mannosidase [22]. The latter finding, confirmed by pulldown experiments under denaturing conditions, indicated that in *T*. *brucei* TL binds to Manβ1-4GlcNAcβ1-4GlcNAc trisaccharide core of Man_5_GlcNAc_2_ paucimannose *N*-glycans [23]. However, the observation that glycopeptides with triantennary complex-type *N*-glycans lacking the LacNAc repeat are not retained on an TL-Sepharose column, suggests that TL blot analysis detects relatively weak interactions between TL and glycoproteins that are not modified by poly-LacNAc [24] and that expose some cryptic glycotopes (*e*. *g*.: paucimannose *N-*glycans) in folded proteins [23].

A few surface receptors have been characterized in *T*. *brucei*, such as those for transferrin (TfR) [25, 26] and haptoglobin-hemoglobin (HpHbR) [27], whereas no receptor and only a few proteins of the endocytic pathway have been fully identified in *T*. *cruzi* to date. Among these proteins are two lysosomal proteases (cathepsin L-like cysteine protease (TcrCATL (cruzipain)) [28], serine carboxypeptidase [29]), a cysteine-protease inhibitor (chagasine) [30], two P-type H^+^-ATPase isoforms (TcHA1 and TcHA2 [31]) and TcRab11 [32]. In *T*. *brucei*, it was initially postulated that proteins belonging to the endocytic pathway (glycosyl-phosphatidyl inositol (GPI)-anchored receptors such as the ESAG6 subunit of the TfR [25, 26, 33]) are posttranslationally modified by poly-LacNAc addition, which has been proposed to act as sorting signal for endocytosis [34]. However, recent data revealed that the TfR subunits ESAG6 and 7 are devoid of poly-LacNAc structures and are modified by oligomannose and paucimannose *N*-glycans, so that their association with glycoproteins bearing poly-LacNAc must allow their binding to ricin and TL [35]. Thus, a direct link between TfR-linked modification and endocytosis can be excluded [35]. Although giant chains of poly-*N*-LacNAc (~ 54 LacNAc repeats /glycan) have been identified as a gel-like matrix able to bind both ricin and TL in the lumen of both the FP and the endosomal/lysosomal compartment [20], inhibition of the synthesis of almost all complex-type *N*-glycans, which includes poly-LacNAc, only marginally affected the *in vitro* growth rate of bloodstream forms in either *TbSTT3A* or *TbSTT3BC* knock-down cells [36]. Albeit these data contrasted with the previous data suggesting a role of poly-LacNAc in the uptake of Tf, LDL and HDL as the latter was significantly reduced with high molar excess of chito-oligosaccharides (chitotriose and chitotetraose) [34], they demonstrated that, at least *in vitro*, *T*. *brucei* does not require poly-LacNAc glycans for receptor-mediated endocytosis [36].

Using three different lectins, TL that is mainly specific to poly-LacNAc units, ricin that is specific to terminal β-D-galactose units and GSLII that specifically binds to the non-reducing terminal residue of *N*-acetylglucosamine (GlcNAc), we evaluated whether *N*-glycans binding to TL and ricin characterize the endocytic components of *T*. *cruzi* as they do in *T*. *brucei*. Both TL and ricin specifically targeted the endocytic compartments of the parasite (mainly cytostome and endosomal network), and GSLII labeled ER structures. In addition, the uptake of Tf, but not of dextran, was inhibited by TL in a process competed out by a molar excess of chitin hydrolysate, suggesting that in *T*. *cruzi* poly-LacNAc glycans and/or paucimannose/oligomannose derived structures are involved in receptor-mediated endocytosis.

## Methods

### Parasite culture

*Epimastigotes*: *T*. *cruzi* epimastigotes (Dm28c, culture collection of Fundação Oswaldo Cruz) [37] were grown in Liver Infusion Tryptose (LIT) (Difco) medium at 28°C [38] and harvested after three to four days of growth (phase log). Around 8 x 10^7^ parasites are equivalent to 1 mg proteins [39]. *Metacyclic trypomastigotes*: Epimastigotes were allowed to differentiate into metacyclic trypomastigotes *in vitro* by incubation under chemically defined conditions [40]. Epimastigote parasites were harvested at saturation (5 days) and centrifuged at 1,500 x *g* for 15 min at 4°C, resuspended at 2 x 10^8^ cells/ml in Triatomine Artificial Urine (TAU) medium (190 mM NaCl, 8 mM phosphate buffer, 17 mM KCl, 2 mM MgCl_2_, pH 6.0), and incubated for 2 h at 37°C. The parasites were then diluted to 5 x 10^6^ cells/ml in TAU3AAG medium (TAU supplemented with 0.035% sodium bicarbonate, 10 mM L-proline, 50 mM sodium glutamate, 2 mM sodium L-aspartate and 10 mM glucose) and incubated for 72 h at 28°C. The relative percentages of metacyclics/intermediate epimastigotes were determined by microscopic examination of parasites. *Tissue culture trypomastigotes*: Metacyclic trypomastigotes were incubated with Vero cells (ATCC) in RPMI (GIBCO) medium supplemented with 2% fetal bovine serum (FBS) (Sigma) for 6 h at 37°C. Cells were then washed 5 times with RPMI and incubated in RPMI supplemented with 2% non-decomplemented FBS in 5% carbon dioxide humidified air at 37°C. Until the fourth day, supernatants containing trypomastigotes were collected and used to re-infect Vero cells and the parasites resulting of this double infection were collected. Around 2 x 10^8^ parasites are equivalent to 1 mg proteins [39]. *Amastigotes*: Amastigote-like forms were obtained from a 9-days old culture of infected Vero cells in axenic conditions. After 3 days, the Vero cells were lysed and the medium centrifuged at 500 x *g* for 10 min to remove cellular debris. Amastigotes were collected by centrifugation at 2,500 x *g* for 10 min at 4°C. Around 2 x 10^8^ parasites are equivalent to 1 mg proteins [39].

### Fluorescence microscopy

Epimastigote forms harvested in log phase were washed with PSG pH 8.0 (2.5 mM NaH_2_PO_4_, 47.5 mM Na_2_HPO_4_, 36.5 mM NaCl, 15% Glucose) at 4°C and fixed in PBS (pH 7.4) containing paraformaldehyde 4% (w/v) for 1h on ice. Cells were then permeabilized with 0.1% Triton X-100 for 10 min and the reaction was stopped with TBS-glycine. Cells immediately resuspended in ice-cold PBS to a final concentration of 5 x 10^6^ cells/ml were settled onto poly-L-lysine-coated glass slides in a humid chamber at room temperature and blocked with 5% BSA in TBS for 1h to prevent non-specific binding. Parasites were labeled with either biotinylated TL (1% BSA in TBS, 1 mM CaCl_2,_ 40 μg/ml biotinylated-TL (Sigma)) or biotinylated ricin (Sigma) and then revealed by streptavidin conjugated to Alexa 488 or Alexa 594 (Molecular Probes). For GSLII, the parasites were labeled with GSLII-Alexa 488 (1% BSA (Roche)) in TBS, 1 mM CaCl_2_, 20 μg/ml fluorescent Alexa Fluor 488 conjugate of GSLII (Invitrogen)) and washed with TBS. Co-localization of GSLII with anti-BiP (kindly provided by J. D. Bangs, University of Wisconsin Medical School, Madison) or anti-TcJ6 polyclonal rabbit antibodies were performed by labeling the parasites with 20 μg/ml of GSLII-Alexa Fluor 488 (1% BSA and 1 mM CaCl_2_) in TBS, in presence of 4 μg/ml of purified anti-TcJ6 IgGs or anti-BiP diluted 1:5,000. Primary antibodies were detected with an Alexa Fluor 594-conjugated goat anti-rabbit IgG (Life Technologies) washed three times in TBS. The slides were rinsed sequentially in 70%, 85% and 100% ethanol before being mounted in PBS, 50% (w/v) glycerol and 4,6-diamidino-2-phenylindole (DAPI) stain (0.1 μg/ml). Images were captured on a Zeiss Axioplan 2 microscope coupled to a CCD camera and processed by ISIS 3 and Adobe Photoshop softwares. The specificity of TL and GSLII was probed by incubating the lectins with 0.2 mM chitin hydrolysate (Vector Laboratories, SP-0090) at 37°C for 30 min prior to their addition to the cells [41]. Similarly the specificity of ricin was assessed by incubating the lectin with a molar excess of galactose (200 mM) at 37°C for 30 min prior to be added to the cells.

### Fluorescence microscopy on live cells in agarose pad

Log phase Dm28c clone cells were incubated with 25 μg/mL DyLight 488 labeled tomato lectin (Vector Laboratories) together with 20 μM protease inhibitor (Mu-Phe-hPhe-FMK, Sigma), for 5 min at 28°C in PSG + 1% BSA. 0.2 mM of chitin hydrolysate was added together or not. 50 μg/mL of transferrin-594 was then added. Cells were harvested and washed after 60 min and mounted on a 1% low melting point agarose pad sealed with rubber glue. Parasites were imaged with an Axioimager M2 widefield fluorescence microscope with a 100x Plan-APOCHROMAT 1.4 objective.

### Flow cytometry

Cells treatments and conditions were similar as for live cells microscopy except transferrin-633 was used instead of transferrin-594 to avoid compensation issues. 20,000 live cells were analyzed based on the gating. One morphological FSC/SSC gate followed by one FSC-H/FSC-A gate for effective singlet isolation was performed. Mean fluorescence intensity (mfi) of the gated cells was measured for 488 labeled-tomato lectin (TL) signal (FITC filter) and transferrin-633 (Tf) (Molecular Probes) signal or Dextran-647 (Molecular Probes) (APC filter). Samples from various conditions were displayed either in histograms or in quadrants.

### Electron microscopy

For immunogold detection by ultrathin cryosectioning, cells were fixed in 3% paraformaldehyde, 0.5% glutaraldehyde, 0.1 M cacodylate buffer (pH 7.2), embedded in 10% gelatin and 2.3 M sucrose, and frozen in liquid nitrogen. Sectioning of frozen samples was done on a Leica EM UC7 ultramicrotome [42]. Sections on carbon-formvar grids were probed sequentially with biotinylated TL, rabbit anti-biotin antibodies (Bethyl Laboratories) and protein A-gold conjugate (5 nm), and finally mounted in methyl cellulose—1% uranyl acetate films. Observations were made on a Tecnai 10 electron microscope (FEI) and images were captured with a Veleta camera and processed with AnalySIS and Adobe Photoshop softwares.

### Western blotting

Parasites were washed twice with PSG at 4°C and lysed in Laemmli buffer to a final concentration of 5 x 10^5^ parasites/μl. 10 μl of total protein extracts were separated on a 7.5% SDS-PAGE gel. Protein transfer was realized onto Hybond-P membrane (Amersham) by electrotransfer in TGM (20% methanol) buffer for 1h at 100V. Membranes were blocked by incubation with 5% skim milk powder in PBS and were then incubated with anti-TcrCATL antibodies (kindly provided by Ana Paula C. A. Lima, UFRJ) in TBS and 3.5% milk powder overnight at 4°C. Secondary antibodies, peroxidase-conjugated monoclonal mouse anti-rat IgG (Serotec), were diluted in TBS buffer 1:5,000, and the bound antibodies were detected by chemiluminescence (Perkin Elmer).

### Lectin blotting

Total parasite protein extracts or CHAPS- and Triton-soluble cell lysate fractions were separated on either a 7.5% SDS-PAGE gel or a 4–12% gradient gel (NuPAGE, Invitrogen). Similarly iron-saturated bovine Tf (Sigma) or glycophorin (Sigma) were separated on a 4–12% gradient gel. Blots blocked with 3% BSA were incubated overnight at 4°C with PBS containing respectively, either 4 μg/ml biotinylated TL or 4 μg/ml biotinylated GSLII in 0.1 mM CaCl_2_ and 2% (w/v) Polyvinylpyrrolidone, followed by two washes TBS with 0.05% Tween 20 and 0.1 mM CaCl_2_. The blots were incubated at room temperature for 30 min with streptavidin peroxidase (Sigma) in 50 mM Tris (pH 7.5) and washed two times with TBS containing 0.05% Tween 20, 0.1 mM CaCl_2_ and once with TBS with 0.1 mM CaCl_2_.

### Transferrin endocytosis

Epimastigotes were taken in log-phase, washed in DMEM + 1% BSA and then incubated in the same medium for 30 min at 28°C. After 15min, 20 μM protease inhibitor (Mu-Phe-hPhe-FMK, Sigma) was added to inhibit the cysteine proteases. The parasites were resuspended to 5 x 10^7^ cells/ml in DMEM + 1% BSA, 20 μM protease inhibitor (Mu-Phe-hPhe-FMK, Sigma) and 50 μg/ml Alexa Fluor 594-conjugated to transferrin (Invitrogen). Cells (5 x10^6^) were incubated for 2, 5, 10 and 30 min at 28°C and washed in ice-cold PSG at 4°C and then immediately fixed in PBS containing 4% (w/v) paraformaldehyde for 1h on ice before examination by fluorescence microscopy. Blocking of endocytosis was performed by incubating cells at 4°C in presence of 50 mM deoxyglucose, 0.02% Azide, and 20 μM protease inhibitor (Mu-Phe-hPhe-FMK, Sigma).

### Poly-LacNAc-glycoproteins enrichment by lectin affinity chromatography

2 x 10^10^ parasites were taken in log phase, washed twice in PSG at 4°C and lysed in 25 mM Tris-HCl (pH7.5), 150 mM NaCl, 1% CHAPS supplemented with protease inhibitor (complete Protease Inhibitor Cocktail, Roche) for 1h at 4°C. The extract was first centrifuged at 16,000 x *g* for 15 min at 4°C and the supernatant was then centrifuged at 120,000 x *g* for 80 min at 4°C. After centrifugation, the supernatant was applied to a column of either TL or GSLII coupled to agarose beads (Vector Laboratories). Binding was allowed overnight at 4°C on a rotating device. The above pellets were washed 3 times with 100 mM NaHCO_3_ buffer (pH 11.0) to disrupt protein-protein interactions. Proteins were then extracted twice with 25 mM Tris-HCl (pH 7.5), 150 mM NaCl, 1% CHAPS and 1% Triton X-114 supplemented with protease inhibitor for 30 min at 4°C and then overnight at -20°C. Extracted proteins were applied to a column of either TL or GSLII coupled to agarose beads overnight at 4°C. Bound glycoproteins were eluted with a chitin hydrolysate (either 0.05 M or 0.02 M in 25 mM HEPES (pH 7.8) for GSLII and TL, respectively) and precipitated with 4 volumes of ice-cold acetone overnight at -20°C. Glycoproteins were then resuspended in Laemmli buffer, separated on a 4–12% gradient NuPAGE gel (Invitrogen) and revealed by SafeStain Coomassie Blue (Invitrogen) in order to detect proteins of sufficient amount for further MS analysis.

### PNGase F treatment

TL-enriched glycoproteins were denatured in 100 mM potassium phosphate buffer (pH 7.8), 1% SDS for 5 min at 95°C. Glycoproteins were then diluted to a final concentration of 100 mM potassium phosphate, 10 mM EDTA, 0.1% SDS, 1% NP-40, 1% β-mercaptoethanol supplemented with protease inhibitors (Roche) and digested with PNGase F (2 units) (Roche) for 4h at 37°C.

### Protein identification by LC-MS^2^

The protein bands from SDS-PAGE were excised, reduced, alkylated, and trypsin digested according to Shevchenko A *et al*. [43]. The resulting peptides were fractionated by nano-flow LC using a 10 cm long × 75 μm ID × 3 μm C_18_ capillary columns connected to an EASY-nLC (Proxeon) in tandem to a Waters Q-TOF Ultima Global mass spectrometer (Waters, Zellik, Belgium). The elution was performed with a flow rate of 300 nl/min with a gradient of 10–50% solvent B for 35 min followed by 50–100% for 15 min (solvent A: 2% ACN /0.1% FA; solvent B: 98% ACN /0.1% FA) and directly analyzed on the Q-TOF. The full MS scan was collected in the positive mode in the mass range from 300–1200 m/z. The three most intense ions (doubly and triply charged ions) were submitted to CID with 15–40 V collision energy. Acquired MS data were processed by Mascot Distiller (v.2.3.2.0) using default settings in order to generate peak lists that can be submitted to database search. Derived peak lists were searched against *T*. *cruzi* protein database (TC_Tcruzi release 6.0, 91482 protein entries: http://tritrypdb.org) using in-house mascot software (matrixscience). Database search parameters were the following: trypsin (lysine and arginine-specific enzyme) as the digestion enzyme (one miscleavage site allowed), 150 ppm for peptide mass tolerance, 0.8 Da for fragments mass tolerance, carbamidomethylation of cysteine residues and oxidation of methionine residues as fixed and variable modifications, respectively. Only proteins that matched to a minimum of two different peptides identified with highly significant database matching scores (p-value of 0.05) were assigned as conclusively identified. Those identified with a single peptide had their spectra manually inspected for the presence of at least three y and b consecutive and most intense ions.

### Bioinformatic analysis of identified proteins

Subcellular localization, GPI and glycosylation were annotated using either literature references or prediction tools. GPI-modification was predicted using FragAnchor (http://navet.ics.hawaii.edu/~fraganchor/NNHMM/NNHMM.html), glycosylation using NetNGlyc (http://www.cbs.dtu.dk/services/NetNGlyc/) and subcellular localization prediction using PSORT II (http://wolfpsort.org/). For hypothetical proteins homologs, a blastp search was performed against the NCBInr database. Only homologs with e-values ≤ 10^−10^ were accepted.

## Results

### Detection of *T*. *cruzi* TL-binding glycoproteins

**Fig 1:**
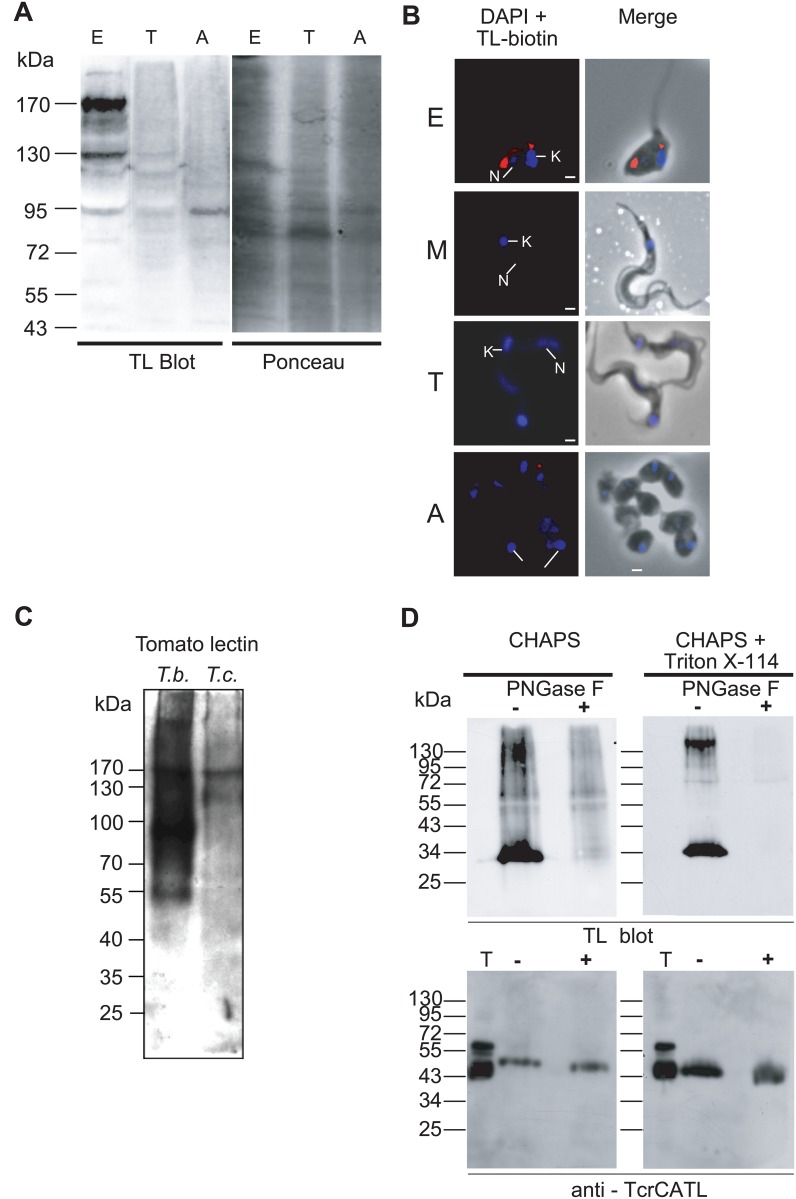
Tomato lectin blotting and fluorescence microscopy analyses. (A) TL blotting on total protein extracts of three developmental forms of *T*. *cruzi*. Similar amounts of proteins (around 50 μg) from three *T*. *cruzi* stages were loaded (see Material and Methods). The same membrane blot was revealed with ponceau red as loading control. The lectin blot analyses indicate that TL-binding glycoproteins are significantly present in epimastigote forms. E: epimastigote, T: trypomastigote, A: amastigote. (B) Fluorescence microscopy of three developmental forms of *T*. *cruzi* probed with biotinylated tomato lectin. Arrows indicate the position of nucleus (N) and kinetoplast (K) stained in blue by DAPI. E: epimastigote; M: metacyclic, T: trypomastigote, A: amastigote. Bars scales represent 2μm. (C) TL blotting on total extract of *T*. *brucei* bloodstream forms (10^6^ cells) vs *T*. *cruzi* epimastigote forms (5 x10^6^ cells). (D) TL blots of *T*. *cruzi* CHAPS- and Triton-soluble (CHAPS+Triton X-114) cell lysate fractions. Fractions were enriched by TL chromatography and then treated (+) or not (-) with PNGase F and T represents the total cell lysate. Blots were either probed with TL (upper panel) or anti-TcrCATL (lower panel). The TL blot indicates the presence of *N*-glycan modification in both soluble and membrane fractions. Treatment of the fractions with PNGase F abolished the reactivity of TL confirming *N*-glycoprotein type modification. The lower panel shows the presence of TcrCATL, a poly-LacNAc-modified glycoprotein, in both fractions. PNGase F treatment results in the appearance of a lower band corresponding to the loss of the N-glycosylation. Apparent molecular weights are indicated in kDa on the left.

To reveal the presence of TL-binding glycoproteins of *T*. *cruzi*, total protein extracts of the parasite were probed with TL. Three main life stages of *T*. *cruzi* were assessed by TL blotting attesting the presence of TL-binding sites in dividing epimastigote forms and to a lower extent in amastigote forms (Fig 1A). The presence of TL-binding sites in dividing epimastigote forms was confirmed using biotinylated-TL followed by streptavidin-conjugated Alexa 594 on fixed cells, but not in quiescent metacyclic trypomastigote or culture trypomastigote forms (Fig 1B). A weak labeling was observed in proliferative amastigote forms suggesting that this latter form could contain few TL-binding sites. These findings suggest that in *T*. *cruzi* TL-binding sites are developmentally regulated and are specifically expressed in the endocytically active epimastigote forms. The amount of TL binding sites in these parasites was much lower than that of the *T*. *brucei* bloodstream form, which contains giant poly-LacNAc [20, 34] (Fig 1C). We next investigated the nature of the glycan linkage by lectin affinity chromatography followed by PNGase F treatment. A similar set of TL-bound proteins, different from that observed in total protein extracts (Fig 1A and 1C), ranging in molecular weight from 30 to over 130 kDa, was found in epimastigote lysate fractions extracted with CHAPS (CHAPS-soluble fraction) and CHAPS + Triton X-114 (Triton-soluble fraction) (Fig 1D, upper panel). Digestion with PNGase F allowed the demonstration that in these proteins poly-LacNAc glycans and/or paucimannose structures are bound via an *N*-glycosidic linkage to asparagine residues. As expected, this protein fraction contained the major lysosomal cysteine protease TcrCATL (previously named cruzipain), which is modified by a sulfated high-mannose oligosaccharide reported to contain 2 to 4 *N*-acetyllactosamine repeats in the glycan part of the C-terminal domain [44] (Fig 1D, lower panel). Whereas two major bands were recognized by an anti-TcrCATL antibody in the total lysate fraction from epimastigotes, likely due to variable posttranslational modifications [45, 46] including carbohydrate heterogeneity [47], only one band of around 53 kDa was detected in the soluble TL-binding fraction (CHAPS) [46]. Upon PNGase F treatment of this fraction the band shifted down to ~ 50 kDa, which corresponded to the expected molecular weight of the amino acid sequence. Interestingly, in membrane-bound enriched fraction (Triton-soluble) the apparent molecular weight of TcrCATL was lower than in the CHAPS-soluble fraction presumably due to the presence of a GPI anchor as previously suggested [48, 49] although TcrCATL does not display clear GPI anchor addition signal.

### *T*. *cruzi* TL- and ricin-binding glycoproteins follow the parasite endocytic pathway

**Fig 2:**
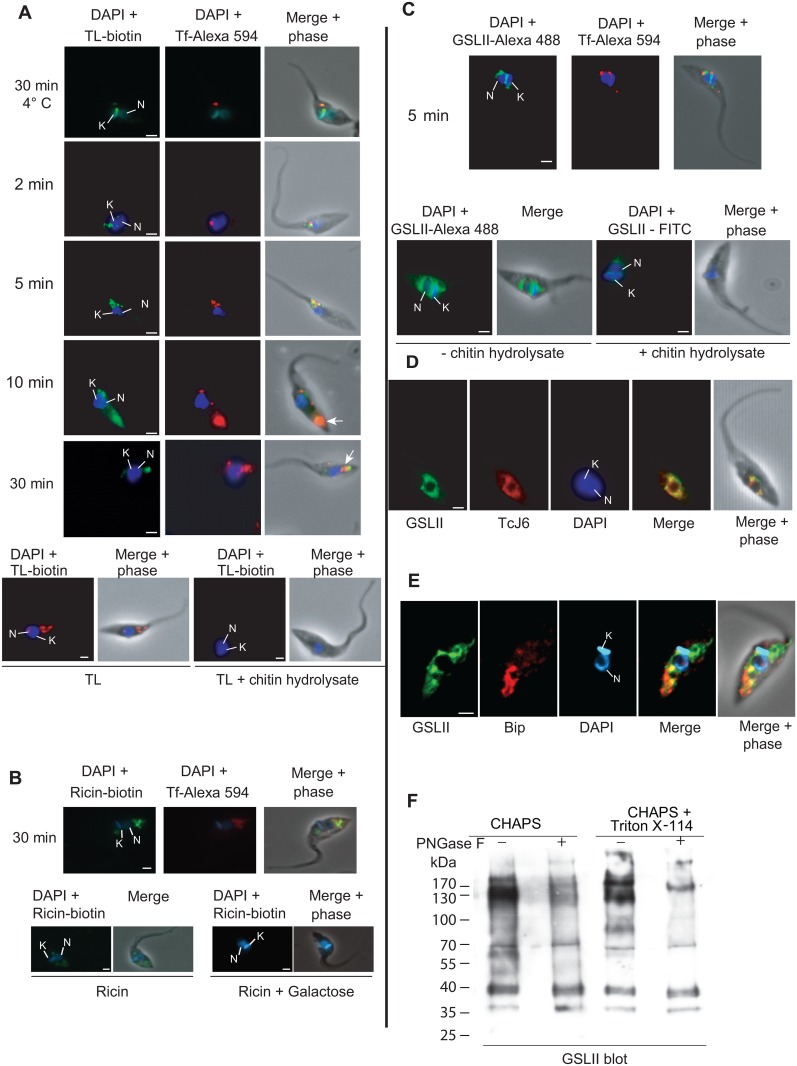
Localization of TL and GSLII binding sites in *T*. *cruzi*. Endocytosis kinetics of fluorescent Alexa Fluor 594 conjugated Tf was performed in order to follow *T*. *cruzi* endocytic pathway from the flagellar pocket/cytostome to the reservosomes. Parasites were fixed at different time points and probed with biotinylated TL (A), biotinylated ricin (B) or Alexa 488 conjugated GSLII (C). The addition of chitin hydrolysate clearly shows inhibition of TL and GSLII staining. (A) Co-localization of biotinylated-TL (green) and Tf (red). (B) Co-localization of biotinylated-ricin (green) and Tf (red). Addition of 200 mM galactose abolished the ricin staining. (C) Co-localization of Alexa 488 conjugated GSLII (green) and Tf (red). (D) Co-localization of Alexa 488 conjugated GSLII (green) and TcJ6 (red). (E) Co-localization of Alexa 488 conjugated GSLII (green) and anti-BiP (red). (F) GSLII blotting of cell extracts enriched by GSLII chromatography. GSLII blots of *T*. *cruzi* CHAPS- and Triton-soluble (CHAPS+Triton X-114) cell lysate fractions were enriched by GSLII chromatography and then treated (+) or not (-) with PNGase F. Blots were probed with biotinylated-GSLII. The GSLII blot indicates the presence of *N-*acetylglucosamine modification in both soluble and membrane fractions. Treatment of the fractions with PNGase F decreased the reactivity of GSLII confirming *N*-glycoprotein type modification.

The cellular fluorescent labeling observed with the endocytic tracer Alexa Fluor 594-conjugated to Tf was compared with that obtained using biotinylated TL bound to streptavidin Alexa 488 (Fig 2A). The specificity of TL binding was assessed by incubating epimastigotes with biotinylated TL in the presence of an excess of chitin hydrolysate, which abolished the TL signal (Fig 2A, bottom). In the absence of Tf uptake (incubation for 30 min at 4°C in presence of deoxyglucose (50 mM) and azide (0.02%)), the green TL labeling was co-localizing as a punctuated signal with Tf in the anterior region of the cell, which presumably corresponds to the cystostome. After 2 min of Tf internalization at 28°C, a Tf signal was still observed in the anterior region of the cell, close to cytostome and FP, partially co-localizing with that of TL. After 5 min, the Tf signal was in the perinuclear region and after 10–30 min it migrated to the posterior part of the cell, where it concentrated into reservosomes that appeared to be equally labeled by biotinylated TL (arrows in Fig 2A). In order to assess the specificity of the TL labeling we used a terminal β-D-galactose-specific lectin ricin, which is supposed to recognize a larger set of glycoproteins including those bound by TL [20, 35]. Similar co-localization of the Alexa Fluor 594-conjugated Tf with the biotinylated ricin bound to streptavidin Alexa 488 was observed along the antero-posterior cell axis (Fig 2B) and the ricin signal was abolished when cells were pretreated with galactose, attesting the carbohydrate binding specificity of the lectin. The observation that TL/ricin labeling localized almost exclusively to the endocytic pathway suggests that poly-LacNAc structures are present in the endocytic compartments (see next section), such was demonstrated for TcrCATL [44]. We next attempted to localize, among the *N*-glycans exposing terminal GlcNAc residues, the precursor forms of poly-LacNAc glycans by using a GlcNAc-binding lectin (GSLII) reported for its ability to recognize exclusively *N*-acetylglucosamine residues on the non-reducing terminal end of oligosaccharides [50]. GSLII staining showed a perinuclear distribution that did not co-localize with Tf, as expected (Fig 2C). No significant staining was observed with cells co-incubated with an excess of chitin hydrolysate (Fig 2C, lower panel) attesting the labeling specificity. The green GSLII signal co-localized with that obtained with an antibody against TCJ6 co-chaperone, a hsp40-like involved in translation initiation, a marker with a punctate pattern distributed throughout the cytosol of the cell, which preferentially concentrated in the perinuclear region [51], suggesting an unexpected ER distribution for GSLII-binding sites (Fig 2D), although we can not exclude a Golgi distribution as well. This result was confirmed using an anti-BiP antibody, which is currently used as marker for ER in *T*. *brucei* (Fig 2E) [52]. To assess the nature of the glycan linkage, GSLII-enriched glycoproteins fractions treated with PNGase F decreased significantly the GSLII-binding indicating that GlcNAc terminal residues are incorporated into N-linked glycoproteins and probably to a lower extent into O-linked glycoproteins (Fig 2F).

### Subcellular localization of TL-binding sites in *T*. *cruzi*

**Fig 3:**
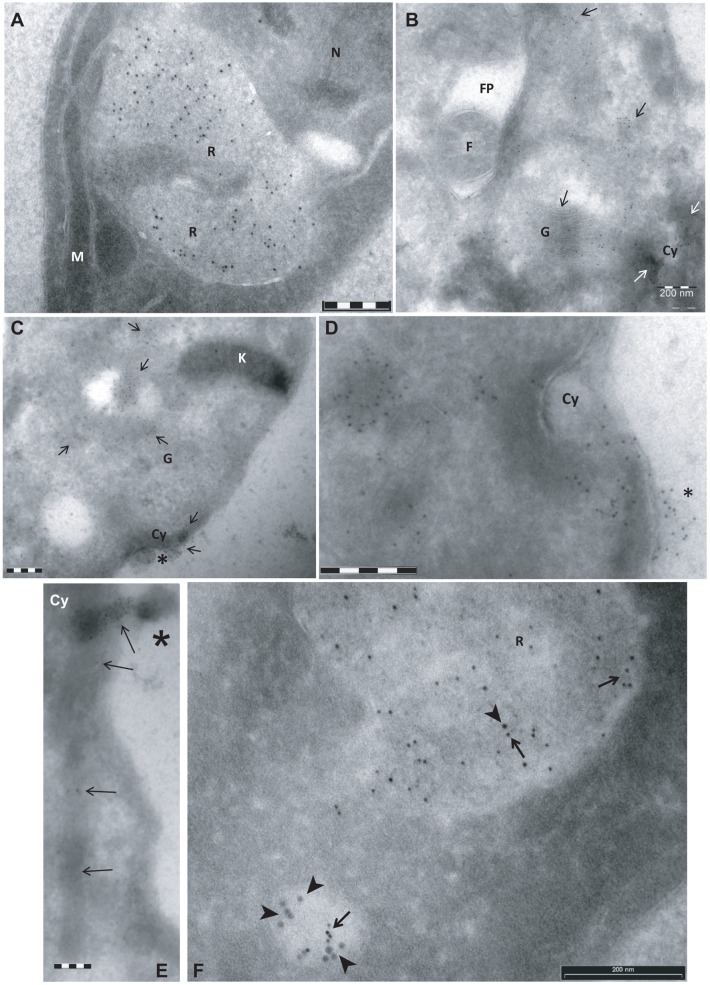
Subcellular localization of TL-binding sites in *T cruzi* by transmission electron microscopy (TEM). Parasites were incubated for 5 min in PSG medium in presence (F) or absence (A-E) of BSA-gold as endocytic tracer (10 nm). Cells were fixed and processed for ultrathin frozen sectioning (Tokayasu method, [42]). Cryosections were sequentially probed with biotinylated TL, rabbit anti-biotin antibodies, protein A-gold (5 nm) and finally mounted in methyl cellulose-uranyl acetate films. Representative images are shown. K: kinetoplast, M: mitochondrion, R: reservosome, N: nucleus, FP: flagellar pocket, F: flagellum, G: golgi, Cy: cytostome. Arrows and arrowhead, point to gold particles that mark the presence of TL binding sites and BSA-gold particles, respectively. Asterisk show TL-binding matrix near the opening of the cytostome. Bars = 200 nm.

In order to investigate the subcellular localization of TL-binding sites in *T cruzi*, a transmission electron microscopy (TEM) TL-gold analysis was performed on ultrathin cryo-sections of epimastigote forms probed with biotinylated TL followed by rabbit anti-biotin Ab and protein-A gold (5 nm). Most TL-binding sites were localized at the posterior end of the cells, in single membrane vesicular structures most probably corresponding to reservosomes (Fig 3A). Significant labeling was also found at the anterior region and close to the kinetoplast. The labeling at this region was found on membrane-bound tubular structures corresponding those of the Golgi apparatus (Fig 3B and 3C) and on early endosomes neighboring the FP, but not in the lumen of the FP (Fig 3B, 3C and 3D). Significant labeling was detected on the electron-dense region corresponding to the cytostome, more prominent at its opening at the cytoplasmic membrane (Fig 3B and 3D). This labeling may correspond to that observed by fluorescence microscopy at the anterior end of epimastigotes (Figs 1B and 2A). At this cytostome entry site, the TL-binding was clearly on an electron-dense diffuse matrix that sometimes protruded out of this organelle (Fig 3C, 3D and 3E; asterisks). Co-localization of BSA-gold (10 nm, arrowhead), used as endocytic tracer with protein-A gold (5 nm, arrow), demonstrated that TL-binding sites are present in vesicular structures belonging to the endocytic apparatus (Fig 3F). Thus, TL-binding-sites are concentrated in the endocytic pathway.

### Identification of TL- and GSLII-enriched glycoproteins of *T*. *cruzi* by mass spectrometry

**Fig 4:**
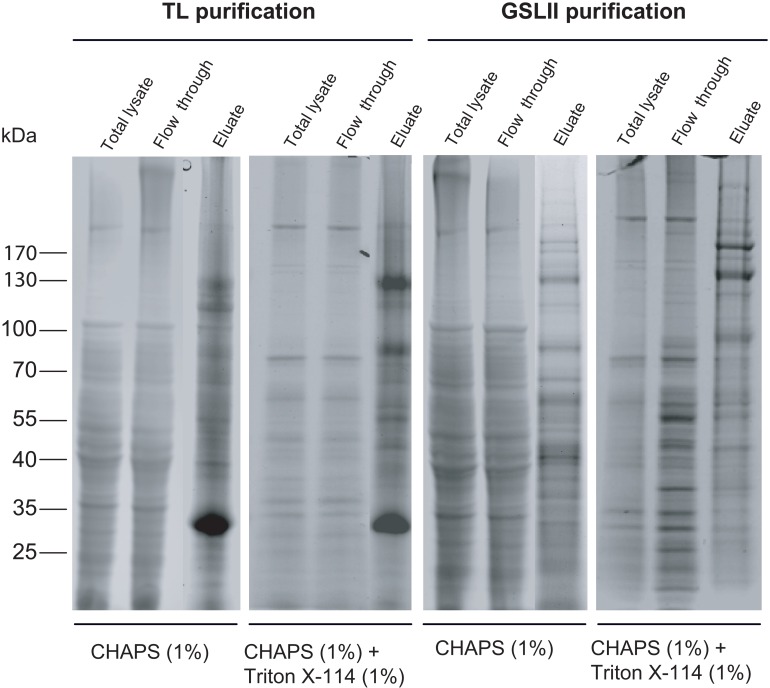
Enrichment of glycoproteins from *T*. *cruzi* epimastigote using TL and GSLII affinity chromatography. *T*.*cruzi* epimastigote proteins were fractionated by detergent extraction into CHAPS and CHAPS + Triton X-114 fractions. These fractions were loaded either onto agarose-coupled TL or GSLII beads columns and left overnight at 4°C on a rotating device. Whole cell extracts, columns flow-through and eluates were then separated on NuPAGE gels (4–12%) and proteins were revealed by SafeStain blue staining.

Assuming that TL labeling localizes almost exclusively to the endocytic pathway, we used a lectin affinity chromatography strategy to characterize endocytic glycoproteins by proteomic analysis. A similar approach was used for the characterization of GSLII binding glycoproteins supposed to characterize a largest spectrum of glycoproteins exposing terminal GlcNAc residues including poly-LacNAc *N*-glycans. Proteins from epimastigote lysates extracted sequentially by CHAPS and CHAPS-Triton X-114, respectively, were applied to either TL or GSLII columns. A clear difference in the protein pattern of TL and GSLII eluates, compared to the total extracts or the flow-through, indicated protein enrichment after lectin chromatography (Fig 4).

**Fig 5:**
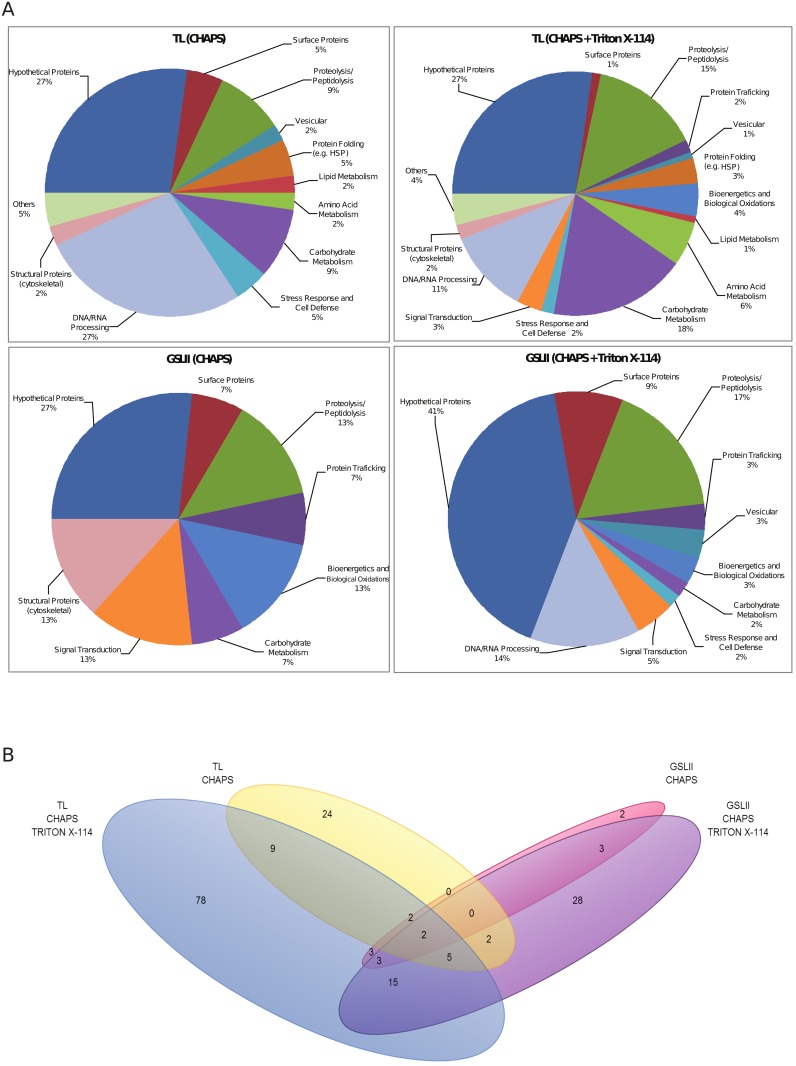
Pie charts (A) displaying protein families function distribution and Venn diagram (B) showing the repartition of the identified proteins in the different lectin-binding fractions. (A) Functional classification of proteins in TL- and GSLII-enriched fractions. The chart shows the different metabolic pathways to which the identified proteins with known or hypothetical function were assigned. The percentages within each group are indicated. (B) Numbers of identified proteins (with the exception of the proteins grouped under others and hypothetical) in TL and GSLII fractions are represented by a 4-tiered Venn diagram indicating the level of protein overlap between the different lectin-binding fractions. Notable regions include protein groups specific to only one lectin-type: blue (TL-CHAPS + Triton) and yellow (TL-CHAPS), violet (GSLII-CHAPS + Triton) and pink (GSLII-CHAPS) as well as groups identified across lectins and fractions (mixed color regions).

TL and GSLII-enriched bands were digested with trypsin and analyzed by LC-MS/MS. Searching against *T*. *cruzi* TriTrypDB protein database (http://tritrypdb.org) resulted in the identification of a total of 234 proteins (S1 Table). Genome annotation of the identified proteins predicted that they are involved in diverse biological processes (S1 Table and Fig 5A) while up to 27% and 41% of TL and GSLII proteins, respectively, were hypothetical with so far unknown functions.

**Fig 6:**
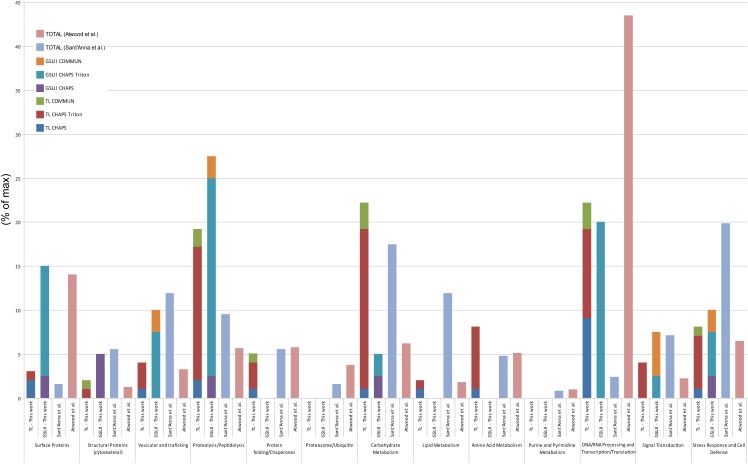
Comparisons of the identified protein families in three independent proteomic studies [53, 54]. The percentages of different protein families identified in three different studies are compared. The stacked bar chart represents the cumulative distribution of the different fractions shown for each protein family. Functional classification of *T*. *cruzi* proteins was performed according to Atwood *et al*. [53]. Proteins grouped under others and hypothetical were discarded from this comparison.

As expected, the comparison of the identified protein families (beyond hypothetical and others) in TL and GSLII subproteome fractions with that of both epimastigote global proteome [53] and reservosome proteome [54] (Fig 6) showed an enrichment of proteins implicated in the endocytic pathway such as proteolysis and peptidolysis (19.1% and 27.5%, respectively, in TL and GSLII subproteomes versus 9.5% in the reservosome and 5.6% in the global proteome).

Enrichment was also observed for vesicular and trafficking proteins (4% and 10% in TL and GSLII, respectively, versus 11.9% in the reservosome and 3.2% in the global proteome), cytoskeletal proteins (5% in GSLII, 5.5% in the reservosome versus 1.2% in the global proteome), proteins of the carbohydrate metabolism (22.2% in TL, 17.4% in the reservosome versus 6.2% in the global proteome), signal transduction (4% and 7.5% in TL and GSLII, respectively, versus 7.1% in the reservosome and 2.2% in the global proteome), and stress response and cell defense (8% in TL, 10% in GSLII, 19.8% in the reservosome versus 6.4% in the global proteome). Proteins involved in the proteasome, lipid metabolism and purine/pyrimidine metabolism were either absent or underrepresented in our work compared to both other studies, while proteins involved in DNA/RNA processing were highly represented in our work, although still less than the global proteome, compared to the reservosome study (22.2 and 20%, respectively, in TL and GSLII, 2.3% in the reservosome and 43.4% in the global proteome). Comparable numbers were obtained in the three studies for chaperones and proteins involved in folding (5.1% in TL, 0% in GSLII, 5.6% in the reservosome and 5.8% in the global proteome). From a total of 176 proteins matched in the two subproteomes (Fig 5B), a subset of 25 proteins were common to both TL and GSLII Triton-soluble fractions, encompassing 6 lysosomal proteins (cysteine proteases including the *bona fide* cruzipain (TcrCATL), a lysosomal alpha-mannosidase and a cytosolic leucyl aminopeptidase), the lysosomal/endosomal membrane protein p67, a glycoprotein molecular marker of the *T*. *brucei* lysosome [41, 54], a vacuolar ATPase found in the subcellular fraction of *T*. *cruzi* reservosomes [54]), 11 hypothetical proteins, and some probable contaminants (3 ribosomal proteins, one protein match for activated protein kinase C receptor, glyceraldehyde-3-phosphate dehydrogenase glycosomal, paraflagellar rod protein 3, ATPase beta subunit and tryparedoxin peroxidase) (S1 Table and Fig 5B). Some additional putative lysosomal proteins were found specifically in the TL membrane-bound fraction, such as the serine carboxypeptidase CBP1, a marker of the *T*. *cruzi* endocytic compartment and reservosomes [29, 55]. Interestingly, most of the proteins identified in TL- and GSLII-binding fractions were found in CHAPS+Triton X-114 extractions (72.7% and 79.4%, respectively), suggesting that most proteins were enriched in the Triton-soluble fractions which correspond to a selectively enriched membrane protein fraction [56].

### Specific inhibition of Tf uptake by TL in *T*. *cruzi*

**Fig 7:**
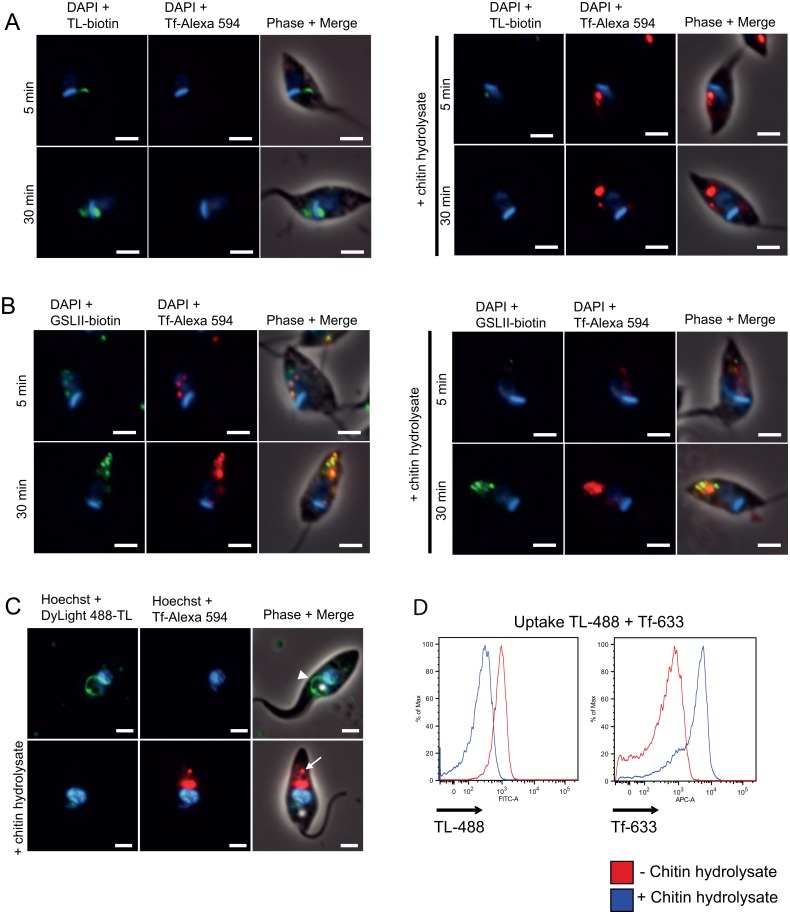
Inhibition of uptake of Tf by TL in epimastigote forms of *T*. *cruzi*. Trypanosomes preincubated with biotinylated TL in the presence of 20 μM FMK-024 (25 μg/ml) and in the absence (A, left panel) or presence of competing chitin hydrolysate (A, right panel), were then incubated with Tf Alexa-594 for 5 or 30 min at 27°C. Cells were then fixed and treated for fluorescence microscopy. Similar incubations wherein TL was substituted by GSLII (B) were performed to assess the specificity of the TL labeling. Furthermore, live parasites preincubated with DyLight 488-TL and 20 μM protease inhibitor (FMK-024) for 5 min and then incubated for 60 min in the presence of Alexa Fluor 594 conjugated Tf showed a lectin labeling in the cytostome/cytopharynx (arrowhead), while no Tf labeling (red signal) was observed in these conditions (C, upper panel). In presence of a molar excess of chitin hydrolysate an intense labeling of Tf exclusively concentrate into reservosomes (arrow) while no green signal corresponding to TL was observed anymore (C, lower panel). Inhibition of trypanosomes Tf uptake with TL was furthermore quantified by flow cytometry (D). The TL signal was dropping from 913 to 273 of mfi in the absence or presence of chitin hydrolysate, respectively (D, left histogram). Conversely, Tf signal was increasing from 597 to 3793 of mfi in the absence or presence of chitin hydrolysate, respectively (D, right histogram).

**Fig 8:**
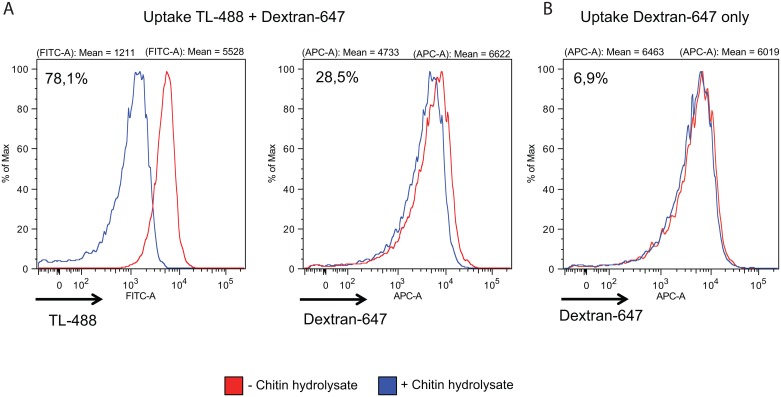
Uptake of Dextran in the presence of TL in epimastigote forms of *T*. *cruzi*. Flow cytometry profiles of uptake of Dextran Alexa-647 by trypanosomes in the presence or absence of biotinylated TL. Trypanosomes preincubated (A) or not (B) with biotinylated TL in the presence of 20 μM FMK-024 (25 μg/ml) and in absence (A, left histogram) or presence of competing chitin hydrolysate (A, right histogram), were then incubated with Dextran Alexa-647 for 30 min at 27°C.

**Fig 9:**
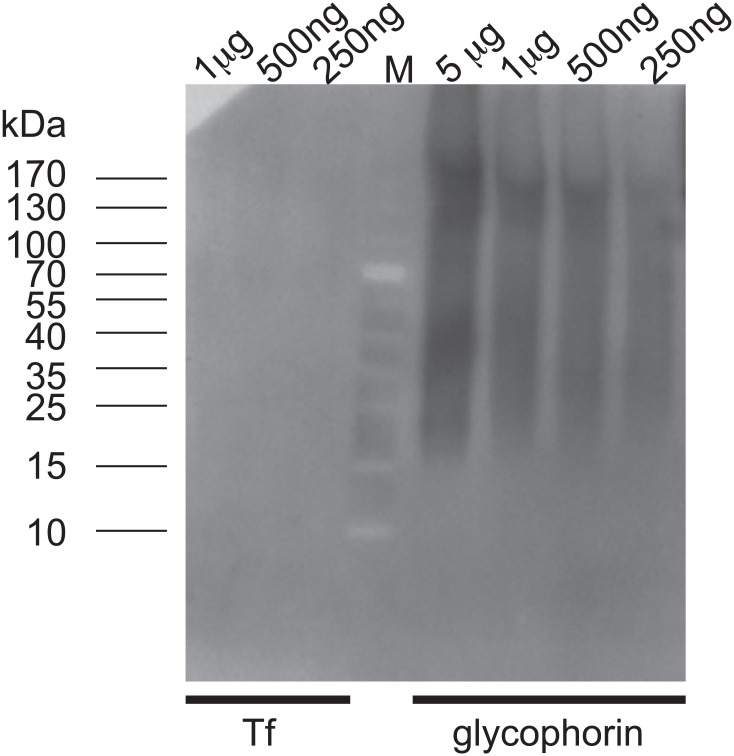
TL blotting on Tf and glycophorin. Different amounts of proteins (up to 5 μg) were loaded. The lectin blot analysis indicates that TL does not recognize Tf but reacts with the sialoglycoprotein glycophorin.

In order to assess the role of TL-binding components in *T*. *cruzi* endocytosis, we incubated live parasites with TL in the presence or not of 0.2 mM chitin hydrolysate as competitor, and followed the kinetics of Tf endocytosis. Remarkably, most of the cells with lectin-bound cytostome/cytopharynx were devoid of Tf, and conversely in the presence of chitin hydrolysate, Tf was taken up in the absence of TL signal (Fig 7A). The use of the GSLII as negative control showed that this lectin did not interfere with Tf endocytosis (Fig 7B). Moreover, the chitin hydrolysate competed for GSLII binding only after short incubation with the lectin (5 min) (Fig 7B). Similar results were obtained using live cells incubated in the presence of TL directly coupled to the fluorochrome (Fig 7C). Quantitative analysis performed by flow cytometry (Fig 7D) revealed that TL inhibited the uptake of Tf by 93.6%, and this inhibition was almost completely reverted in the presence of chitin hydrolysate, linked to 70% decrease of TL uptake (S1 Fig). In order to assess whether TL is also able to inhibit fluid endocytosis, we incubated live *T*. *cruzi* cells with TL in the presence or not of chitin hydrolysate as competitor, and quantify by flow cytometry the Dextran endocytosis (Fig 8A). TL did not affect Dextran uptake in conditions where TL binding was highly effective. While addition of chitin hydrolysate dropped down TL binding by 78.1%, uptake of Dextran decreased by 28.5%. This slight decrease of the fluid phase cargo is not directly linked to the competition by chitin oligosaccharides alone (6,9%) (Fig 8B) and probably results from a steric hindrance. We finally checked the possibility that Tf, which in the case of human Tf contains two major glycosylation sites harboring mainly bi- and triantennary complex type glycans, could directly bind TL (Fig 9). Whereas lectin blot analysis showed a clear reaction with different amounts of glycophorin used as positive control with biotinylated TL, no reaction was observed for Tf even with high amount of protein (up to 1 μg) [57]. This result strongly suggests that inhibition of Tf uptake by TL does not result from a direct lectin interaction with the ligand.

## Discussion

### Cytostomal endocytosis versus flagellar pocket endocytosis

Much of the research on the molecular process of endocytosis in trypanosomatids has been focused on African trypanosomes [6], and has shown that essential growth factors are rapidly internalized via the FP through highly specific receptors coupled to a polarized endocytic pathway with an extremely high rate of traffic and sorting [25–27]. This situation contrasts with that of the closely related species, the Stercorarian South American trypanosome, which probably diverged from the Salivaria parasites about 100 million years ago [58]. This parasite exhibits several peculiarities in its endocytic/exocytic pathway that distinguishes it from *T*. *brucei*, such as the presence of a cytostome, a surface area dedicated to macromolecule ingestion, where the plasma membrane invaginates deeply into the cytosol as a funnel-like structure forming the cytopharynx [59]. Hence, endocytosis in *T*. *cruzi* is mostly concentrated on a smaller surface area at the bottom of the cytopharynx [7, 8, 60, 61], although it was recently shown that the “naked” side of the cytopharynx, devoid of underlying microtubules, possesses endocytic activity [3]. The cargo is finally delivered via the tubulo-vesicular endosomal system to reservosomes, specialized terminal lysosomes where ingested macromolecules are stored [9]. Our ultrastructural analyses of epimastigote forms indicated that TL-binding sites were associated with the endocytic compartment (cytostome/cytopharynx structures, early endosomes and reservosomes), as an electron-dense diffuse matrix of glycoproteins similar to that found in both the FP and the endosomal lumen of bloodstream African trypanosomes [25, 62]. We also found a very weak signal of biotinylated-TL in amastigote intracellular parasites, which possess a cytostome-like structure, but apparently lack endocytosis [15, 63]. In TEM, no TL-binding sites were found in the lumen or limiting membrane of the FP. The observation that ricin-binding sites specifically locate in similar endocytic structures (*e*. *g*., reservosomes) suggests that TL labeling restricted to endocytic compartments/Golgi may be specific to poly-LacNAc units [20, 35]. These observations suggest that complex modification of *N*-glycoproteins, possibly by poly-LacNAc chains (*e*. *g*., TcrCATL, [44]), is linked to entry sites where endocytic activity is very intense (FP in bloodstream African trypanosomes versus cytostome in *T*. *cruzi* insect-stage). However, whereas in *T*. *cruzi* this *N*-glycans modification is stage-specific, lysosome-related organelles of the endocytic pathway are present in all parasite stages [63]. Therefore, it appears that even in the absence of such *N*-glycans modification, the localization of endocytic markers (TcrCATL, chagasin and serine carboxypeptidase) through the endocytic pathway is kept unaltered in absence of detectable endocytic activity.

### Elusive role of poly-LacNAc modification in endocytic trafficking of trypanosomes

As observed for the *T*. *brucei* lysosomal type I membrane glycoprotein p67 (analogous to LAMP in mammals) [41], in *T*. *cruzi* the poly-LacNAc modification of *N*-glycans, as reported for TcrCATL, probably takes place in the Golgi apparatus. However, GSLII labeling suggested that addition of GlcNAc might also occur early in the ER. Accordingly, a dolichol-independent *N*-acetyglucosaminyltransferase was isolated from both ER and Golgi of trypanosomatids [64] suggesting that in *T*. *cruzi*, GlcNAc might be added to nascent side-chains of *N*-glycans very early during the glycoprotein biosynthetic process. This observation is reminiscent of the intracellular apicomplexan parasites, such as *Plasmodium falciparum*, where GSLII labeled ER, rhoptries, and surface of plasmodia, and no apicoplast [65]. LAMP proteins have been identified as the major carriers for poly-LacNAc in many eukaryotic cells. Among these proteins, the ubiquitous LAMP-1 and LAMP-2, which are structurally and functionally related, are the major components of the lysosomal membrane (reviewed by [66, 67]). It was proposed that the poly-LacNAc chains attached to lysosomal membrane glycoproteins might play a critical role in maintaining the protein stability in lysosomal compartment [68, 69]. Similarly, the giant poly-LacNAc structures harbored by some LAMP-related glycoproteins in *T*. *brucei* (*e*. *g*., the membrane protein p67 analogous to LAMP) may form a continuous coat on the inner surface of the lysosomal membrane that serves as a barrier to soluble hydrolases. However, in *T*. *cruzi* the number of poly-LacNAc repeats harbored by *N*-glycoproteins, such as identified in TcrCATL (~ 2–4 repeats/glycan), might be considerably lower in comparison with *T*. *brucei* (~ 54 repeats/glycan). This view is consistent with the observation that *T*. *cruzi* epimastigote forms contain much less TL-binding sites than *T*. *brucei* bloodstream forms. Interestingly, short poly-LacNAc chains (on average ~ 5 repeats) of the surface lipoglycan from in *Trichomonas vaginalis* were shown to be involved in parasite binding to host cells arguing that similar short chains can play functional role in protein binding [70]. In *T*. *cruzi*, short poly-LacNAc linear chains that transit between the cell surface and the endocytic apparatus might be involved in other biological processes such as glycoprotein trafficking/sorting of lysosomal proteins. Nolan *et al*. hypothesized that in *T*. *brucei* bloodstream form, poly-LacNAc chains might act as sorting signals in the endocytic pathway via a poly-LacNAc recognition by a putative lectin-like receptor [34]. In this respect, our data did not show any effect of chitin hydrolysate on either Tf or dextran endocytosis in *T*. *cruzi* ruling out the role of a lectin-binding glycoprotein in protein sorting and trafficking in this organism. In addition, poly-LacNAc may also play a role as a tag for targeting *N*-glycoproteins to trypanosomal lysosomes because these organisms lack the classical cation-independent mannose-6-phoshate pathway for delivery of soluble proteins bearing the mannose 6-phosphate modification to lysosomes [71, 72]. However, it is very unlikely that poly-LacNAc modification of *N*-glycans was involved in routing of glycoproteins to the lysosomes in *T*. *cruzi* because the localization of endocytic glycoproteins continues unaltered during all cell cycle whereas poly-LacNAc modification appears to be stage-specific [63].

### A possible role of lectin-sugar interactions in trypanosomal endocytic efficiency?

In *T*. *brucei* bloodstream forms, knock-down of components involved in *N*-glycan biogenesis, either TbSTT3A or TbSTT3BC oligosaccharyltransferase, led to a severe *in vivo* growth phenotype, while *in vitro* growth was only slightly affected, suggesting that N-glycosylation could be required for parasite growth in the host [36]. This phenotype may be linked to the arrest of poly-LacNAc modification of *N*-glycans as shown by the significant decrease in ricin-binding sites upon RNAi induction. Therefore, non-optimal endocytosis might be critical *in vivo*, but not *in vitro*, as was also reported following mild alteration of endocytic recycling via rab11 knock-down [73]. In addition, two observations suggest that poly-LacNAc *N*-glycan modification might be involved in endocytosis of *T*. *brucei*: (i) in the presence of high concentration of competing chitin hydrolysate the uptake of Tf is reduced of about 5-fold [34]; (ii) the binding of TfR to TL, whether directly through paucimannose *N*-glycans [23] or indirectly via others glycoproteins [35], could explain the observed accumulation of this receptor in the lumen of the FP [74], and account for the presence of electron-dense matrix in this lumen [25]. Such local concentration of ligand-loaded receptors would obviously improve the efficiency of ligand uptake, possibly more important under *in vivo* than *in vitro* growth conditions. This mechanism might be important for parasite-host fitness allowing the trypanosome to optimize growth and feed efficiency. Our EM observations showing the presence of TL-binding sites associated to an electron-dense matrix in the *T*. *cruzi* cystostome, the functional analog of the *T*. *brucei* FP, are in favor of this model. Moreover, our finding that in *T*. *cruzi* TL strongly inhibits Tf uptake in a process reverted by competing chitin hydrolysate, further points to functional linkage between *N-*glycan modification of endocytic components and ligand uptake. Because of the elusive nature of the *T*. *cruzi* TfR, we cannot postulate on the mechanism by which the parasite internalizes its ligand but the observation that dextran uptake is unaffected by TL highly suggests that complex and/or paucimannose *N*-glycans are involved in a RME specific process, which is not linked to a fluid-phase mechanism.

### Endosomal origin of TL-binding proteins

TL-binding fraction of *T*. *cruzi* did not contain highly abundant surface glycoproteins such as members of the transialidases superfamily (Tc-85), mucin-like glycoproteins or MASPs. This could be due either to the low abundance of these proteins in the epimastigote form as reported by Atwood *et al*. [53] and/or to the extensive O-glycosylation of these proteins (~ 60% of their molecular mass) preventing them from tryptic digestion [75]. Both TL and GSLII proteomes of *T*. *cruzi* contained ~ 80% of putative *N*-glycoproteins (TL and GSLII fractions, CHAPS and Triton fractions, 81%, 79%, 81% and 77%, respectively). This percentage is significantly higher than *N*-glycoprotein encoded in eukaryotic genomes (64% of putative proteins containing the sequon NXS/T in SWISS-PROT protein database [76]), suggesting an enrichment in putative *N*-glycoproteins. In comparison with the global *T*. *cruzi* proteome [53], endosomal/lysosomal proteins represented around 23–37% versus 8.8%, which is nearly similar to that found in the sub-proteome of the reservosome [54]. Several of them were not detected in the whole proteome studies such as the vacuolar ATPase (V0 complex subunit D (TcCLB.508397.10) and a V1 subunit A of vacuolar ATPase (TcCLB.503929.10) which could be involved in endosome acidification, although it has been reported that in *T*. *cruzi* this role is played by P-type ATPases [31]. In this respect, we found a P-type ATPase isoform (TcCLB.505763.19) already identified in the reservosome analysis and the global *T*. *cruzi* proteome (TcHA2) [54]. Other proteins specifically detected in our proteome as the carbonic anhydrase-like protein (TcCLB.508817.130; TcCLB.509597.20) and the cytosolic leucine aminopeptidase (TcCLB.509859.40) were also identified in the proteome of *T*. *brucei* bloodstream plasma membrane [77]. Trypanosomal carbonic anhydrase might localize in endocytic compartments as already described in higher eukaryotes [78]; in addition, this protein was also detected in GPI-anchored proteins-enriched fraction from *T*. *cruzi* epimastigote ([75], S1 Table). Finally, several hypothetical proteins that we identified were either found in the proteome of the reservosomes (TcCLB.511283.290; TcCLB.506637.20; TcCLB.511759.30, TcCLB.510329.10, TcCLB.509683.10, TcCLB.507711.200, TcCLB.509647.13, TcCLB.510099.20) or were specific to our proteome (*i*. *e*., not found in the global proteome), and might constitute interesting candidates as endocytic glycoproteins for further studies (TcCLB.506239.20, TcCLB.503855.50, TcCLB.510717.40, TcCLB.437121.9, TcCLB.509857.60, TcCLB.506303.80, TcCLB.507641.50, TcCLB.508777.110, TcCLB.508175.100, TcCLB.510299.10, TcCLB.511231.69, TcCLB.510889.170, TcCLB.510421.130) as illustrated by the identification of a C-type lectin-like (TcCLB.503855.50), which is homologous to lectin-like mannose receptors. This protein could interact with carbohydrate-containing *N*-glycoproteins of the host, similarly to a lectin-like scavenger receptor. Interestingly, some enzymes of the carbohydrate metabolism were highly represented in our TL subproteome such as a hypothetical protein (TcCLB.511759.30) sharing 98% identity with an UDP-GLcNAc:polypeptide *N*-acetylglucoaminyltransferase from *T*. *cruzi*. Its presence is not unexpected because these enzymes could be trapped and purified together during the glycan biosynthesis wherein poly-LacNAc residues are added to linear side-chains of *N*-glycan in the Golgi apparatus. In the proteomic analyses of TL-binding fractions several proteins not strictly related to endocytic compartments were detected. Most of them are ribosomal proteins (~ 22%) or are components of the cytoskeleton, flagellum, mitochondrion, glycosome, or acidocalcisome. Despite stringent pellet washing with alkaline sodium carbonate buffer we did not succeed to reduce the level of hydrophobic membrane-associated proteins in the Triton fractions. Finally, the presence of contaminants is probably due to their high abundance as deduced from their high mascot scores in the global proteome (S1 Table) [53]. Among them, we found structural protein (α-tubulin), several stress proteins (hsp85, tryparedoxin peroxidase), proteins involved in energetic metabolism (pyruvate phosphate dikinase 1, glutamate dehydrogenase, enolase, glyceraldehyde-3-phosphatase dehydrogenase, D-isomer specific 2-hydroxyacid dehydrogenase), and DNA/RNA processing (poly(A)-binding protein and ribosomal proteins).

## Main Conclusions

Our study suggests that similar *N*-glycan modifications of proteins belonging to the endocytic pathway occurs in *T*. *cruzi* epimastigotes as it does in *T*. *brucei*, with however a major difference: in *T*. *cruzi* these modifications are involved in Tf endocytosis, which takes place mainly at the level of the cytostome, representing the major entry site for endocytosis. Although the nature of the *T*. *cruzi* TfR remains elusive, we propose that in this parasite, lectin-sugar interactions, such as those involving potentially poly-LacNAc modification of *N*-glycans, are implicated in receptor-mediated endocytosis of macromolecules, such as Tf. In addition, we showed that blockade of Tf uptake by TL is not directly mediated by the presence of an endogenous glycan modification of the cargo itself suggesting that Tf internalization could be indirectly mediated via some trypanosome glycoproteins harboring *N-*glycans required for Tf binding. Further characterization of these *N*-glycans in *T*. *cruzi* would allow getting insights into the molecular mechanisms by which the parasite internalize its ligand, which could constitute an unexpected point of attack against the Chagas disease.

## Supporting Information

S1 Fig Representative flow cytometry profiles of the TL-488 and Tf-633 signals in *T*. *cruzi*. Full representation of Fig 7D experiment (representative experiment) showing the gating and the quadrant plots of the TL signal (ordinate) and the Tf signal (abscissa) from live cells treated under different conditions. UC represents the uncolored cells.
(EPS)

S1 Table List of LC-MS/MS identified proteins of TL- and GSLII-enriched fractions from *T*. *cruzi*. Detailed legend is reported on the table.
(XLS)
